# Drivers and potential distribution of anthrax occurrence and incidence at national and sub-county levels across Kenya from 2006 to 2020 using INLA

**DOI:** 10.1038/s41598-022-24589-5

**Published:** 2022-11-22

**Authors:** Valentina A. Ndolo, David William Redding, Isaac Lekolool, David Mumo Mwangangi, David Onyango Odhiambo, Mark A. Deka, Andrew J. K. Conlan, James L. N. Wood

**Affiliations:** 1 Disease Dynamics Unit, Department of Veterinary Medicine, University of Cambridge, Madingley Rd, Cambridge, Cambridgeshire UK; 2 Department of Veterinary Services, Kenya Wildlife Service, Nairobi, Kenya; 3 State Department for Livestock (Kenya), Directorate of Veterinary Services, Kabete, Kenya; 4 Department of Biochemistry, University of Nairobi, Nairobi, Kenya; 5 Department of Genetics, Evolution and Environment, Centre for Biodiversity and Environment Research, University College London, London, UK; 6 US Centers for Disease Control and Prevention, 1600 Clifton Rd. NE, Atlanta, GA USA

**Keywords:** Ecological epidemiology, Ecological modelling

## Abstract

Anthrax is caused by, *Bacillus anthracis*, a soil-borne bacterium that infects grazing animals. Kenya reported a sharp increase in livestock anthrax cases from 2005, with only 12% of the sub-counties (decentralised administrative units used by Kenyan county governments to facilitate service provision) accounting for almost a third of the livestock cases. Recent studies of the spatial extent of *B. anthracis* suitability across Kenya have used approaches that cannot capture the underlying spatial and temporal dependencies in the surveillance data. To address these limitations, we apply the first Bayesian approach using R-INLA to analyse a long-term dataset of livestock anthrax case data, collected from 2006 to 2020 in Kenya. We develop a spatial and a spatiotemporal model to investigate the distribution and socio-economic drivers of anthrax occurrence and incidence at the national and sub-county level. The spatial model was robust to geographically based cross validation and had a sensitivity of 75% (95% CI 65–75) against withheld data. Alarmingly, the spatial model predicted high intensity of anthrax across the Northern counties (Turkana, Samburu, and Marsabit) comprising pastoralists who are often economically and politically marginalized, and highly predisposed to a greater risk of anthrax. The spatiotemporal model showed a positive link between livestock anthrax risk and the total human population and the number of exotic dairy cattle, and a negative association with the human population density, livestock producing households, and agricultural land area. Public health programs aimed at reducing human-animal contact, improving access to healthcare, and increasing anthrax awareness, should prioritize these endemic regions.

## Introduction

Anthrax is a bacterial disease caused by *Bacillus anthracis*, a soil-borne bacterium that infects predominantly grazing animals^1^. *B. anthracis* is classified by the National Institute of Allergy and Infectious Diseases (NIAID) as a ‘category A’ pathogen, known to pose the greatest threat to national security, together with the causative agents of plague, botulism, smallpox, and viral hemorrhagic fevers^2^. The bacteria usually exist in the soil as dormant, persistent spores that can infect susceptible animals during grazing^1,3,4^. Although certain species of animals are more susceptible than others, e.g., cattle compared to sheep and goats^5^, even within these susceptible groups vaccinated animals are less likely to become sick post consumption of the bacteria, and therefore consumption does not guarantee infection^1^. Following infection, the spore changes into a vegetative form that releases toxins and multiplies rapidly, often leading to death in susceptible animals^4^. There are three forms of human anthrax disease depending on the transmission route, these include cutaneous anthrax (most common and least lethal form; about 95% of all human cases), gastrointestinal anthrax, and inhalational anthrax (most rare and most lethal; 45% of case fatality rate even with treatment)^1^. Injection anthrax cases observed among heroin drug users, was also recently added to the list^6^. Livestock, wildlife, and human anthrax cases have been documented globally across Africa, Europe, the United States, Australia, and Asia^7^. However, Africa has been reported to have the highest prevalence of anthrax disease in livestock, with a pooled prevalence of 29% based on studies of the prevalence of anthrax in cows, sheep, goats, and buffalo species worldwide^8^.

Incidences of anthrax outbreaks are associated with ecological and socio-economic factors that drive the emergence of the disease^4,7^. Some well-studied ecological associations include precipitation, temperature, soil properties (calcium, pH, moisture, mineral composition), vegetation, host density, and elevation^4,7,9,10^. Socio-economic drivers, on the other hand, have not been well studied despite the evidence of an association between certain human activities (cultivation, land clearance, and irrigation) and livestock anthrax outbreaks^1^. Kenya reported a sharp increase in livestock anthrax cases from the year 2005, with 12% of the sub-counties (decentralised administrative units used by Kenyan county governments to facilitate service provision) accounting for almost a third of the livestock cases^11^. There was a significantly higher risk of livestock anthrax outbreaks in medium and high potential agro-ecological zones compared to the arid and semi-arid low potential zones^11^. A majority of livestock anthrax outbreaks in Kenya tended to occur between January to March (hot and dry season) and April to June (wet and cool season), with fewer outbreaks observed between July to September (dry and cool season) and October to December (the hot and wet season)^11^.

Ecological niche modelling (ENM) applies machine learning algorithms to study the association between species occurrence and the environmental conditions of a given location to estimate the areas that are suitable for the species across a wider geographical location^12^. ENM has been used to model the spatial distribution of *B. anthracis* suitability across the world^7,9,10,13–15^. Although recent studies of *B. anthracis* suitability in Kenya have applied conventional algorithms that provide useful insights^16,17^, these methods are limited due to their inability to cope with random effects such as spatial and temporal pseudo-replication which are common features of most ecological datasets^18^. Without a complete understanding of the true incidence and drivers of anthrax, it is difficult to conduct proper surveillance, diagnosis, prevention, treatment, and control.

Models that can deal with spatial and temporal dependency structures are usually more computationally demanding and mathematically complex^18,19^. However, the recently developed Bayesian hierarchical modelling technique, Integrated nested Laplace approximation (INLA), offers a fast and accurate approach for estimating posterior distributions for such complex models^20^. This methodological development enables us to develop the first Bayesian models that can address the limitations of past ENM studies in Kenya discussed above (principally spatial and temporal pseudo-replication). We use R-INLA to analyse a long-term spatiotemporal dataset of livestock anthrax case data, collected systematically over a surveillance period of 15 years in Kenya. We first develop a purely spatial model to explore associations between anthrax incidence across Kenya and ecological covariates. We then build a spatial–temporal hurdle model (comprising two models: an occurrence and an incidence model) using the same data to investigate the socio-economic drivers of the geographical distribution of anthrax occurrence and incidence at the sub-county level. The occurrence model investigates the factors that determine whether an outbreak occurs or not (presence or absence), while the incidence model investigates what determines the severity of an outbreak (number of livestock cases) after it occurs. Both are equally important because they provide insight that can help policy makers to design interventions to prevent the occurrence of an outbreak or to reduce the severity in the event that it occurs.

## Methods

### Data sources

We analyzed records of confirmed and suspected livestock deaths attributed to anthrax occurring from 1 January 2006 to 31 December 2020 across Kenya (available online along with full code for the analysis in this paper https://github.com/spatialmodels/Kenyan_anthrax_model). The case records covering the entire country were reported from the Kenya Directorate of Veterinary Services (KDVS) located in Nairobi and the five Regional Veterinary Investigation Laboratories located in Nakuru, Eldoret, Karatina, Kericho, and Mariakani. The anthrax outbreaks were considered as any livestock (cattle, goats, sheep, pigs, camels) or wildlife deaths confirmed through clinical and laboratory diagnosis. Clinical diagnosis was defined as an acute disease accompanied by sudden death, bleeding from body orifices, swelling, lack of rigor mortis, and oedema of the neck and face in pigs. Laboratory confirmation was done through methylene blue staining to identify the characteristic bacterial capsule and the rod-shaped bacilli in clinical specimens collected from the infected carcasses.

We extracted data from old paper records of livestock anthrax cases into Microsoft Excel. These records comprised the location of the livestock outbreaks, name of the farmer, number of animals dead and herd size, species affected, date, method of diagnosis, and the details of the reporting veterinary doctor. Since the locations of livestock anthrax outbreaks were reported at sub-county/district levels (districts refer to the old naming given to current sub-counties before the rollout of the current constitution), we recorded the geographic coordinates of livestock cases at the district level. During data cleaning, we removed duplicate coordinates, outliers, and entries with missing variables. In the end, we had 540 livestock cases that we used for analysis. The spatial granularity and prolonged surveillance period of these data allow for a more detailed perspective on the major drivers of anthrax across Kenya. We also collected wildlife data from the Kenya Wildlife Service (KWS). Most of the data from KWS was lacking information on the geographic coordinates of the outbreaks, so we visited the actual locations and collected the coordinates. We recorded 20 wildlife cases that we used to validate the performance of the spatial model.

### Processing socio-economic and ecological covariates

We gathered geospatial data on ecological and socio-economic correlates of *B. anthracis* ecology and distribution. For the spatial model, we obtained the following variables: rainfall, vegetation, elevation, distance to permanent water bodies, and soil patterns. For the spatiotemporal models, we used human population estimates (total population, population density, and male and female population per sub-county), host population (livestock producing households, total number of indigenous, exotic dairy, and exotic beef cattle per sub-county), and agricultural practices that lead to soil disturbance (agricultural area under cultivation, number of farming households, and crop-producing households).

We chose seven environmental covariates for the spatial model based on known correlates of *B. anthracis* suitability identified from previous peer-reviewed studies^9,10,13,15,21–23^. These comprised three soil variables, including soil pH (× 10) in H_2_O at a depth of 0 cm, exchangeable calcium at a depth of 0–20 cm, and soil water availability (volume of water per unit volume of soil) retrieved at a resolution of 250 m from the International Soil Reference and Information Centre (ISRIC) data hub (https://data.isric.org/geonetwork/srv/eng/catalog.search#/home). We used the shallowest depth available because although the bacterial spores can persist in the surface soil for up to five years and indefinitely in much deeper soils^24^, the spores in the surface soils are more likely to trigger host infection^25^. We retrieved monthly Enhanced Vegetation Index (EVI) data from 1 January 2006 to 31 December 2020 (180 tiles in total) from The Aqua Moderate Resolution Imaging Spectroradiometer (MODIS) Vegetation Indices (MYD13A3 v.6) at a resolution of 1 km^2^ (https://lpdaac.usgs.gov/products/myd13a3v006/). The mean EVI was then calculated using QGIS by averaging all 180 tiles. EVI reduces variations in the canopy background and retains precision over dense vegetation conditions. Monthly Climate Hazards Group InfraRed Precipitation with Station data (CHIRPS) rainfall data from rain gauge and satellite observations was retrieved from the United States Geological Service (USGS) at a resolution of 0.05 degrees (https://climateserv.servirglobal.net/map). Since the rainfall data also comprised 180 tiles, the mean rainfall was calculated by averaging all 180 tiles using QGIS. We also collected data on the distance to permanent water bodies from a global hydrology map obtained from ArcGIS version 10.6.1.^26^ and elevation data at 1 km^2^ resolution from the Global Multi-resolution Terrain Elevation Data (GMTED2010) dataset available from USGS (Table 1).

**Table 1:** Summary of the environmental variables used in the spatial model including variable name, unit, and spatial resolution.

Variable name	Units	Spatial resolution
Rainfall	ml	0.05°
Enhanced Vegetation Index (EVI)	units	1 km
Elevation (m)	m	1 km
Distance to permanent water bodies (km)	km (Euclidean distance)	1 km
Soil calcium	cmolc/kg	250 m
Soil pH	Units (0–14)	250 m
Soil water	v%	250 m

For the spatiotemporal sub-county-based models, we accessed the population data per sub-county (total population, male population, female population, and population density) from the 2019 Kenyan census report provided via the Humanitarian Data Exchange platform (https://data.humdata.org/dataset/kenya-population-per-county-from-census-report-2019). We also obtained data on livestock population (numbers of exotic dairy and beef cattle, and indigenous cattle), area of agricultural land in hectares, number of farming households, and the number of households actively practicing agriculture (crop production and livestock production) aggregated to the sub-county level from the 2019 Kenya Population and Housing Census volume IV provided by the OpenAfrica platform (https://open.africa/dataset/2019-kenya-population-and-housing-census).

**Figure 1:**
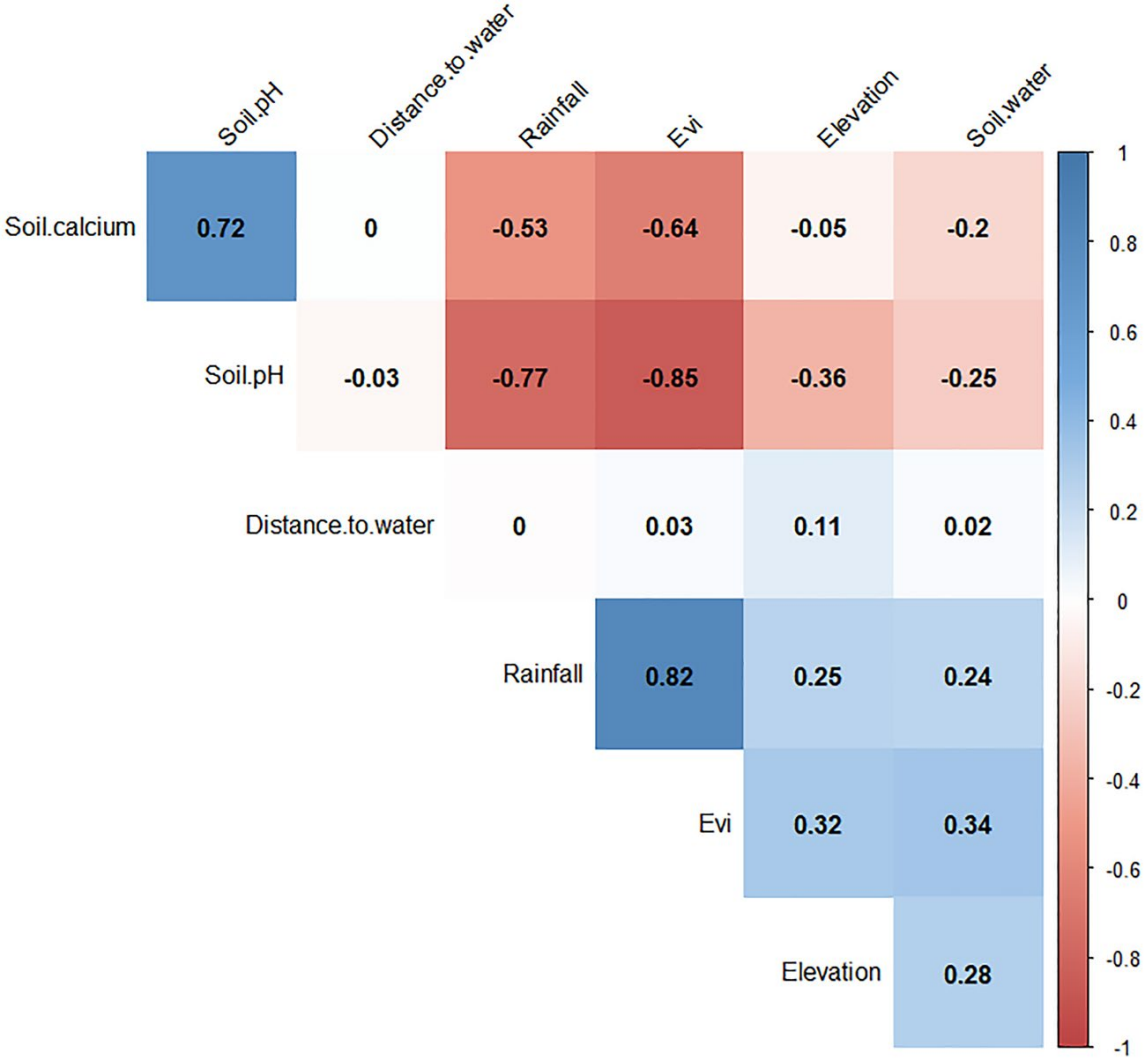
Results of correlation between covariates using Pearson’s correlation coefficient test for the spatial model. Correlation between covariates is shown by red numbers (negative correlation) and blue numbers (positive correlation). Correlations with a p-value > 0.01 are regarded as insignificant and the correlation coefficient values are left blank. The figure was generated using R software v. 4.1.0^28^.

**Figure 2:**
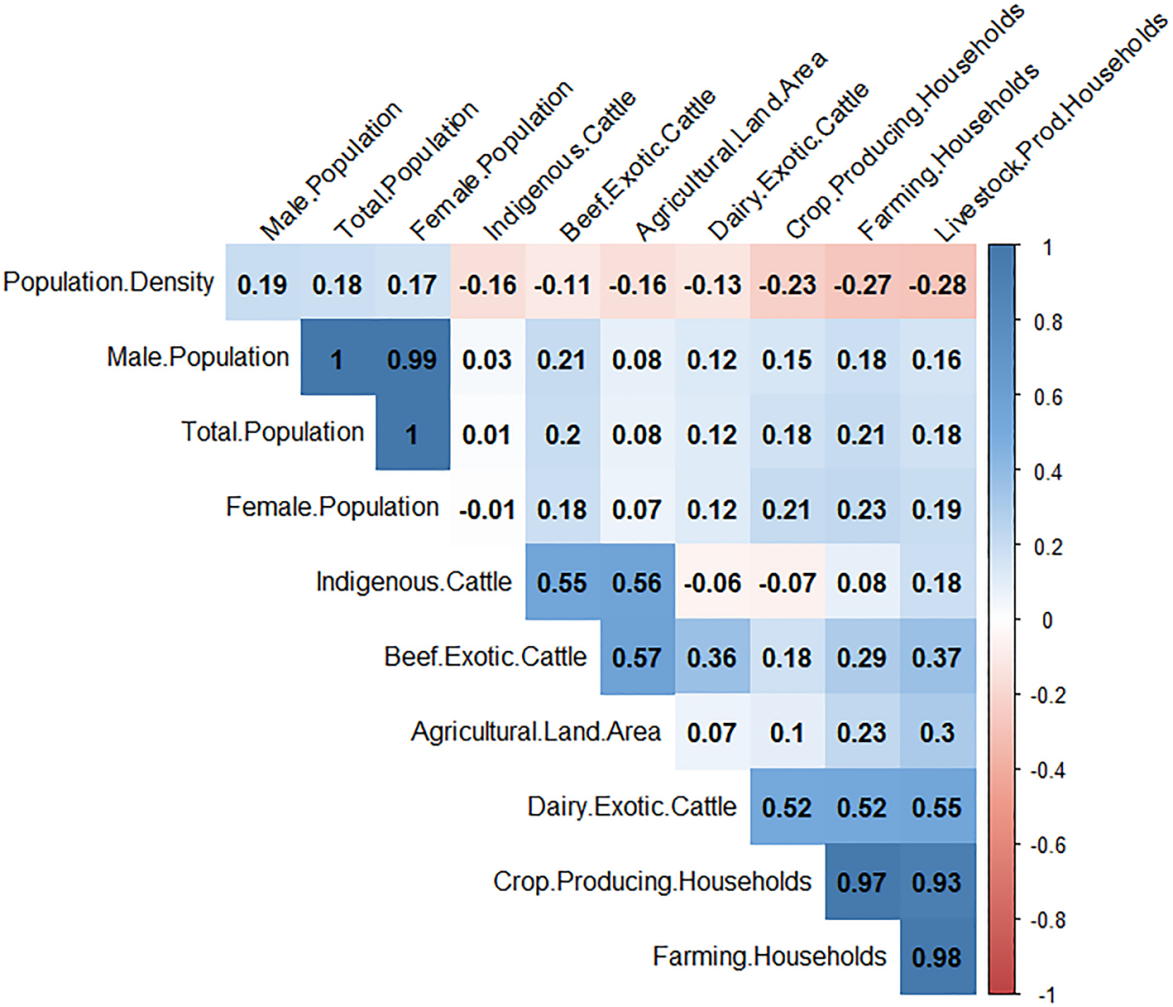
Results of correlation between covariates using Pearson’s correlation coefficient test for the spatiotemporal model. Correlation between covariates is shown by red numbers (negative correlation) and blue numbers (positive correlation). Correlations with a p-value > 0.01 are regarded as insignificant and the correlation coefficient values are left blank. The figure was generated using R software v. 4.1.0^28^.

We conducted data exploration to check for outliers, collinearity, and the relationships between the covariates and the response variables. We used Cleveland dot plots to check for outliers. We measured collinearity using variance inflation factors (VIF), Pearson correlation coefficients, and pairs plots. For VIF scores, the covariates with scores higher than 3 were eliminated one-by-one until all the scores were equal to or less than 3. All the covariates included in the study had correlation coefficient values of less than 0.6 (Figs. 1, 2).

### Spatial model analysis

**Figure 3:**
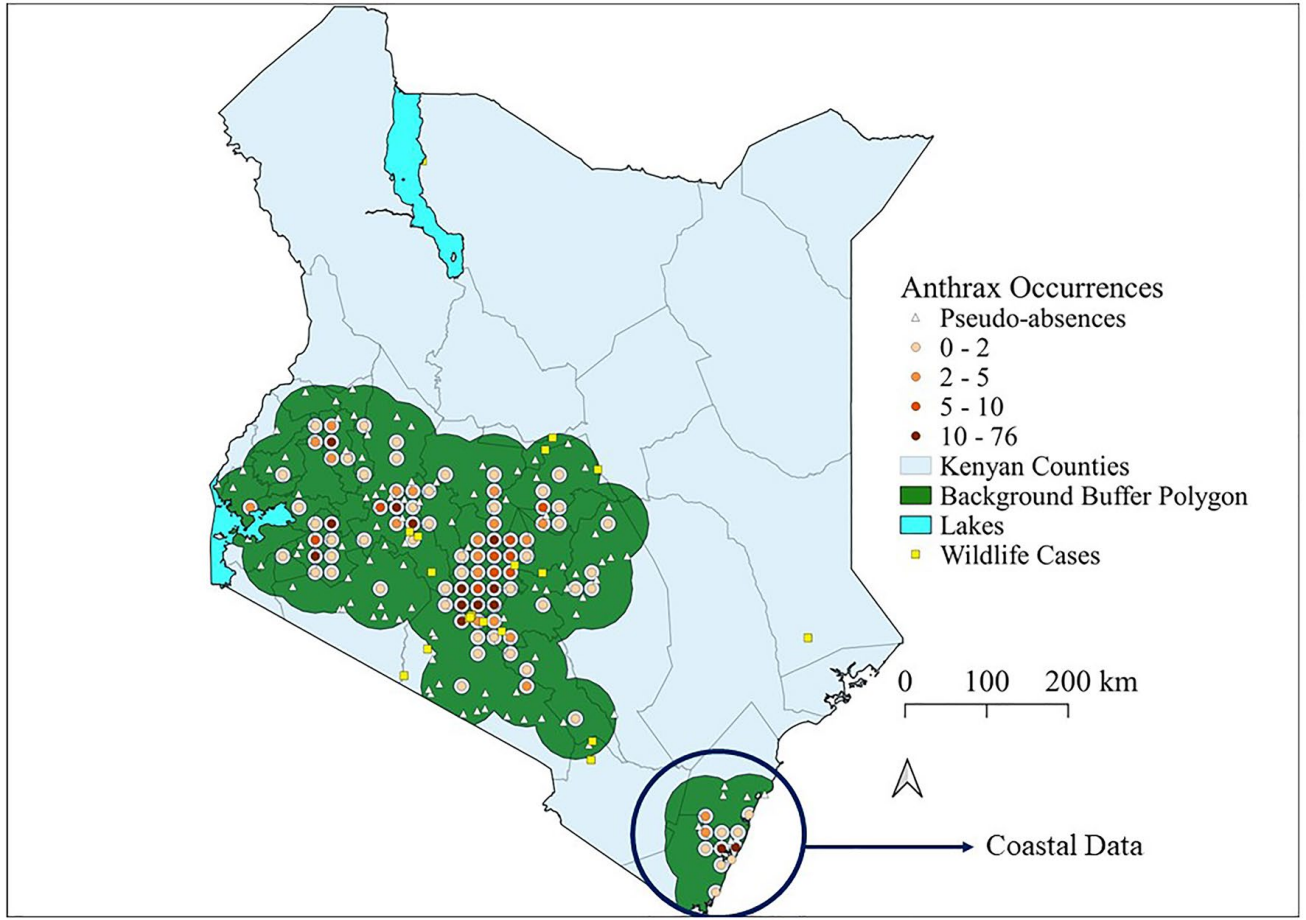
Spatial distribution of thinned livestock anthrax case locations across Kenya from 2006 to 2020. The map shows livestock anthrax case locations (n = 540) thinned to pixels of 20 km^2^ to form 95 new marked locations. The orange dots show the new presence locations which are marked points with colour intensity representing the number of livestock cases per location. The white triangles show the random pseudo-absence locations. The yellow squares are the wildlife cases obtained from the Kenya Wildlife Service. The green polygon is the background calibration buffer used to derive the random pseudo-absence locations. This map was generated using Quantum Geographical Information Systems (QGIS) v. 3.16.11 (https://www.qgis.org/en/site/forusers/download.html).

We used R version 4.1.0 together with the packages raster version 4.1.1^27^, and R-INLA version 4.1.1^28^ to conduct the data processing and statistical modelling. The R-INLA package applies the INLA framework in designing models. We used Quantum Global Information System (QGIS) version 3.16 (https://qgis.org) to create a 50 km buffer polygon around all the observed livestock outbreak points. We then created a 20 km^2^ grid within this buffer and counted the number of points within each grid cell to create a regular lattice with a given number of counts per cell. We then extracted the coordinates of the centroids of each cell to create marked locations with a given number of livestock cases per location. We essentially converted the data into a count process (number of livestock outbreaks per location). We had 95 cells with one or more counts which formed our new presence locations. We then randomly selected 95 pseudoabsences within the 50 km buffer polygon but at a distance of 10 km from the presence locations as shown in Fig. 3.

We defined a Zero-inflated Poisson (ZIP) regression model with spatially correlated random effects, implemented as a generalized additive model (GAM) with anthrax incidence as the response variable. The model is defined as shown in Eqs. (1), (2), and (3)

1 $${C}_{i} \sim zero-inflated\, Poisson \left({\mu }_{i},{p}_{i}\right),$$

2 $$expected\left({C}_{i}\right)=\left(1- {p}_{i}\right)\times {\mu }_{i},$$

3 $$\mathrm{log}\left({\mu }_{i}\right)= \alpha + \sum_{j}{\beta }_{j}{X}_{j,i}+ \sum_{k}{\delta }_{k,i}+{u}_{i},$$

where $Ci$ denotes the observed number of anthrax livestock cases at location *i*_*,*_ ${\mu }_{i}$ and ${p}_{i}$ are parameters of the ZIP distribution. $expected\left({C}_{i}\right)$ refers to the expected number of outbreaks at location *i*, $\alpha$ is the intercept, $\beta$ are the beta coefficients for the covariates, X is the matrix with all the covariates, $\delta k$ are the non-linear effects (cubic regression splines), and ${u}_{i}$ is the spatial random effect at location *i*.

To test whether the addition of the GAM smoothers and the spatially correlated random effects improved the fit of the model, we also considered candidate models without smoothers and spatial random effects. We tested three versions of the spatial model: the first used distance to water, elevation, and EVI as linear covariates without spatial random effects, the second applied non-linear terms to elevation and EVI also without spatial random effects, and the final model was similar to the second model but with the addition of spatial random effects. We then measured the DIC values of the candidate models to select the final spatial model.

We conducted model validation by assessing the posterior distributions of the parameters and the residuals for adherence to the distributional assumptions. We checked whether the residuals were independent and normally distributed. We also plotted a sample variogram to check for any residual spatial auto-correlation using a well-defined method^29^. We then ran 1000 simulations to check whether the model was capable of handling zeros.

The estimated model was used to map posterior predicted distributions for the incidence of anthrax disease (plotted as mean and 95% credible intervals). We validated the model using independent evaluation data withheld from the model calibration. This evaluation dataset comprises the wildlife cases collected from KWS. We then calculated the sensitivity by estimating the proportion of wildlife case locations correctly identified by the model, using the minimum presence training threshold (minimum value of the fitted presence training points).

### Spatiotemporal model analysis

Our second objective was to investigate the socio-economic, population-based drivers of livestock anthrax risk at the sub-county level. These socioeconomic variables are usually collected at the sub-county level. Therefore, we developed a second areal model with the number of observations per sub-county as the new response variable. The occurrence data, gathered by the Kenya Directorate for Veterinary Services (KDVS), consisted of monthly case reports of livestock anthrax cases collected by all 290 sub-counties across Kenya between January 2006 to December 2020. We analyzed the whole monthly case time series from the year 2006 to 2020 and mapped out the annual counts of confirmed and suspected livestock anthrax cases across Kenya at the sub-county level to analyse the spatial and temporal trends throughout the surveillance period. The sub-county shapefiles that were used for mapping and modelling were derived from Humanitarian Data Exchange version 1.57.16 under a Creative Commons Attribution for Intergovernmental Organisations license (https://data.humdata.org/dataset/ken-administrative-boundaries).

Due to the sparsity of data, we aggregated the monthly case counts and modelled the quarterly occurrence and incidence of anthrax at the sub-county-level scale, including spatial and temporal effects, to determine the spatial socio-economic drivers of livestock anthrax disease risk across Kenya. We used R-INLA version 4.1.1 (26) to conduct the data processing and statistical modelling. We used quarterly case counts that were confirmed per sub-county across the 15 years of surveillance (2006–2020) as a measure of anthrax incidence. Due to the zero-inflated and over-dispersed nature of the distribution, which is difficult to fit incidence counts, we employed a two-stage modelling approach using the hurdle model distribution to separately model anthrax occurrence (presence or absence) across all sub-counties via logistic regression, and incidence counts using a zero-inflated Poisson distribution. We were then able separately to estimate the contributions of the various socio-ecological factors that drive disease occurrence (the presence or absence of anthrax) and total incidence counts.

We model the quarterly anthrax occurrence (n = 290 sub-counties over 60 quarters; 17,400 observations) where ${Y}_{i,t}$ refers to the binary presence (denoted as 1) or absence (denoted as 0) of anthrax in sub-county *i* during year *t*, and ${P}_{i,t}$ is the probability of anthrax occurrence, thus:

4 $${Y}_{i,t} \sim Bernoulli\left({P}_{i,t}\right).$$

We model quarterly anthrax incidence counts ${C}_{i,t}$ using a zero-inflated Poisson process with parameters ${\mu }_{i,t}$ and ${p}_{i,t}$ (see Eq. (5)). Equation (6) denotes the expected values for the ZIP distribution at sub-county *i* during year *t*.

5 $${C}_{i,t} \sim Zero-inflated\, Poisson \left({\mu }_{i,t},{p}_{i,t}\right),$$

6 $$expected\left({C}_{i,t}\right)=\left(1- {p}_{i,t}\right)\times {\mu }_{i,t}.$$

Both the Bernoulli and the ZIP distributions are modelled separately as functions of the covariates and the spatial and temporal random effects using a general linear predictor as shown in Eqs. (7) and (8):

7 $$logit \left({P}_{i,t}\right)= \alpha + \sum_{j}{\beta }_{j}{X}_{j,i}+{u}_{i,t}+{v}_{i,t}+{y}_{i,t},$$

8 $$\mathrm{log}\left({\mu }_{i,t}\right)= \alpha + \sum_{j}{\beta }_{j}{X}_{j,i}+{u}_{i,t}+{v}_{i,t}+{y}_{i,t},$$

9 $${y}_{i,t}= {y}_{i,t-1}+ {w}_{i,t},$$

where α denotes the intercept; $X$ signifies a matrix made up of the socio-economic covariates accompanied by their linear coefficients denoted as $\beta$; spatiotemporal reporting trends at the sub-county level were accounted for in the models using spatially structured (${u}_{i,t}$; conditional autoregressive) and unstructured noise (${v}_{i,t}$; i.i.d—independent and identically distributed) random-effects specified jointly as a Besag–York–Mollie model^30,31^, as well as temporally structured (${y}_{i,t}$) random effects of the first order where ${w}_{i,t}$ is a pure noise term that is normally distribute with a mean of zero and a variance of σ^2^. We used uninformative priors with a Gaussian distribution for the fixed effects and penalized complexity priors for the hyperparameters of all the random effects.

For the two spatiotemporal models, we applied linear effects for all the variables: population density, total population, number of exotic dairy cattle, agricultural land area, and number of livestock producing households. We scaled the continuous covariates by standardizing them (to a mean of 0 and standard deviation of 1) before fitting the linear fixed effects.

We used R-INLA to conduct model inference and selection and used DIC to evaluate the model fit for both the occurrence and incidence models. For both models (occurrence and incidence), we created 4 candidate models, compared them, and selected the model with the lowest DIC as the final model. The candidate models included: a baseline intercept only model; a second model with the intercept and covariates; a third model with the intercept, covariates, and the spatial random effects; and a fourth model with the intercept, covariates, spatial random effects, and a temporal trend.

We evaluated the posterior distributions of the parameters and the residuals for adherence to the distributional assumptions. We assessed the residuals to check whether they were independent and normally distributed. We also plotted the residuals against the covariates to check for any non-linear patterns using a well-defined method^29^. We then ran 1000 simulations to check whether the model was capable of handling zeros.

### Ethics statement

Licence to conduct the research was granted by the National Council for Science, Technology, and Innovation (NACOSTI) under reference number 651983, and the Kenya Wildlife Service under reference number KWS-0003-01-21.

## Results

### Spatial model

The best spatial model with the lowest DIC had the distance to water as a linear effect and EVI and elevation as non-linear terms with the addition of spatially correlated random effects. The model also passed validation checks (Table 2).

**Table 2:** Comparison of the three candidate models of the spatial model.

Model	DIC	WAIC
Zero-inflated Poisson Generalized Linear Model (GLM)	1280	1425
Zero-inflated Poisson Generalized Additive Model (GAM)	1057	1258
Zero-inflated Poisson Generalized Additive Model (GAM) + spatial random effects	605	622

**Figure 4:**
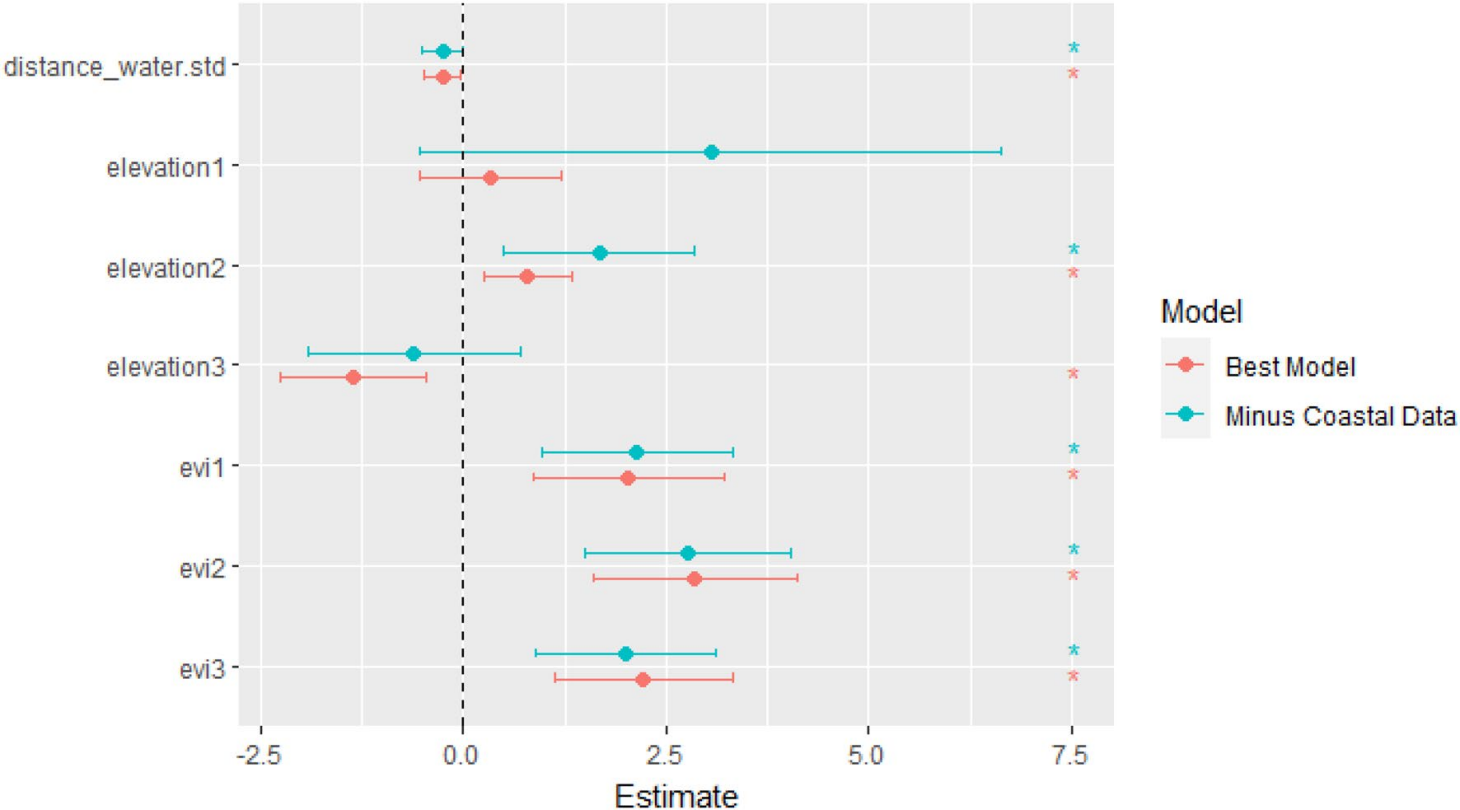
Geographical cross-validation tests for spatial model robustness. The figure shows the magnitude and direction of the fixed effects for the final model and the holdout model to test whether the model was robust following the exclusion of a spatially distinct block of data.

We validated the performance and robustness of the model using both sensitivity of the prediction against withheld data and geographical cross validation. The wildlife cases (n = 20) from KWS were then used to calculate the sensitivity of the model by extracting the predicted values of known wildlife case locations and comparing them against the positivity threshold (minimum training presence threshold) to identify locations that were correctly predicted. The sensitivity was then calculated by dividing the number of correctly predicted test locations by the total number of positive test locations, resulting in a spatial sensitivity of 75% (95% CI 65–75). Geographical cross-validation was done by examining the sensitivity of the model outputs to geographically based cross-validation by fitting a separate model holding out all the livestock data points from the coastal region of the country (n = 11). The fixed effects magnitude and direction were similar for the final model and the holdout model, showing that the findings were robust following the exclusion of a spatially distinct block of data (Fig. 4).

**Figure 5:**
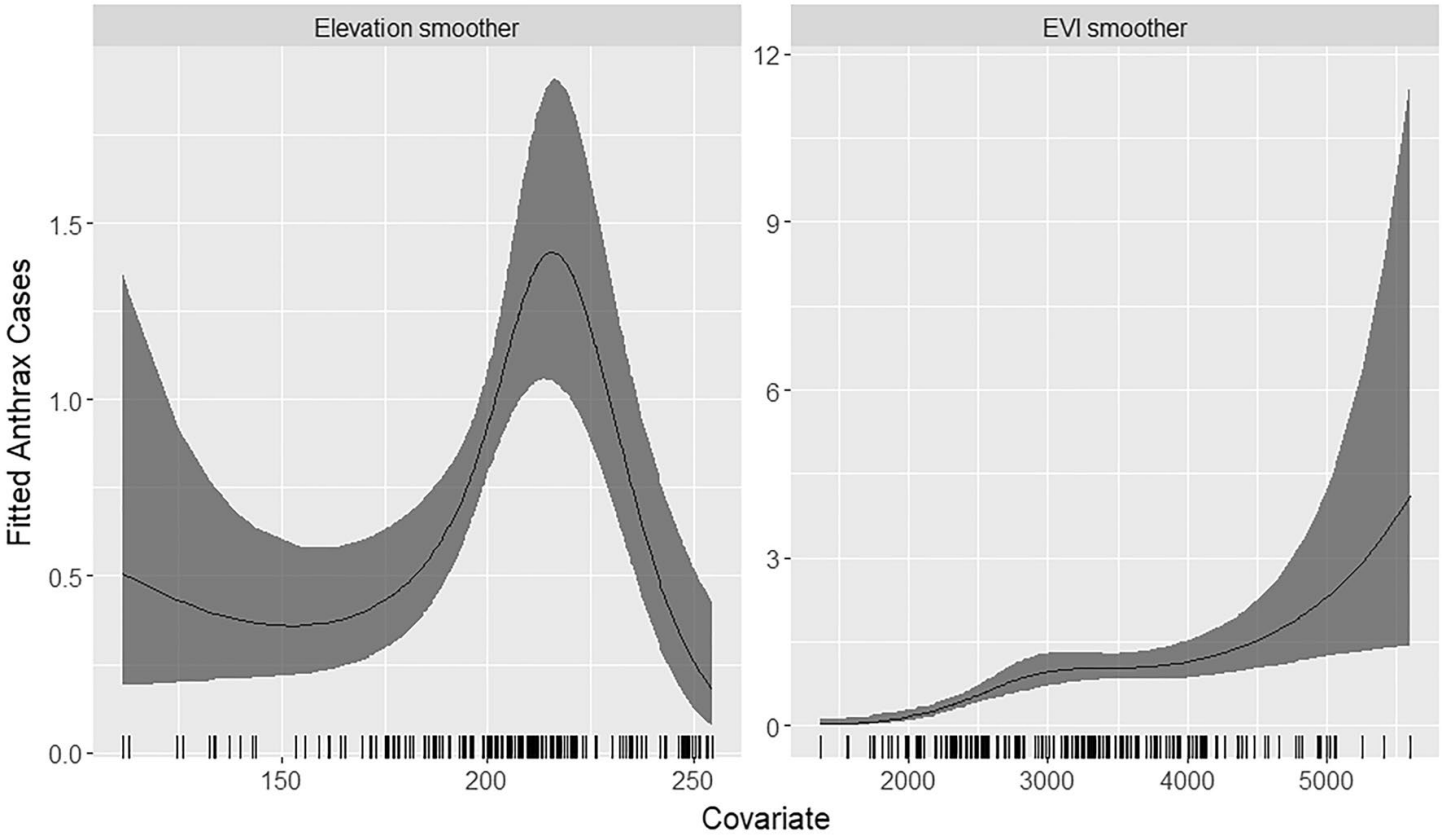
Smoothed fits for Enhanced Vegetation Index (EVI) (units) and elevation (m). The solid black line shows the posterior mean of the smoothing function, and the shaded grey areas represent the 95% credible intervals. The y-axis shows the estimated incidence of anthrax. The figure was generated using R software v. 4.1.0^28^.

The posterior means and the 95% credible intervals for the fixed effects parameters used in the final model are shown in Table 3. The estimated model demonstrates a negative association between distance to permanent water bodies and anthrax incidence. However, EVI had a strong positive association with anthrax incidence between 1367 and 5600 units (Fig. 5). Similarly, increasing elevation was also associated with an increased incidence of anthrax disease from about 150 to 215 m (Fig. 5). The final model was used to predict anthrax incidence across Kenya. The figure was generated using R software v. 4.1.0^28^.

**Table 3:** The posterior means and the 95% credible intervals for the fixed effects parameters of the covariates used in the spatial model.

Variable	Basis functions	Mean	Lower 95% CI	Upper 95% CI
Distance to water (km)		− 0.26^a^	− 0.49	− 0.03
Elevation (m)	1	0.34	− 0.54	1.22
	2	0.80^a^	0.26	1.33
	3	− 1.36^a^	− 2.26	− 0.45
Enhanced Vegetation Index (EVI)	1	2.04^a^	0.87	3.21
	2	2.85^a^	1.60	4.11
	3	2.22^a^	1.12	3.33

^a^Statistically significant posterior mean value.

**Figure 6:**
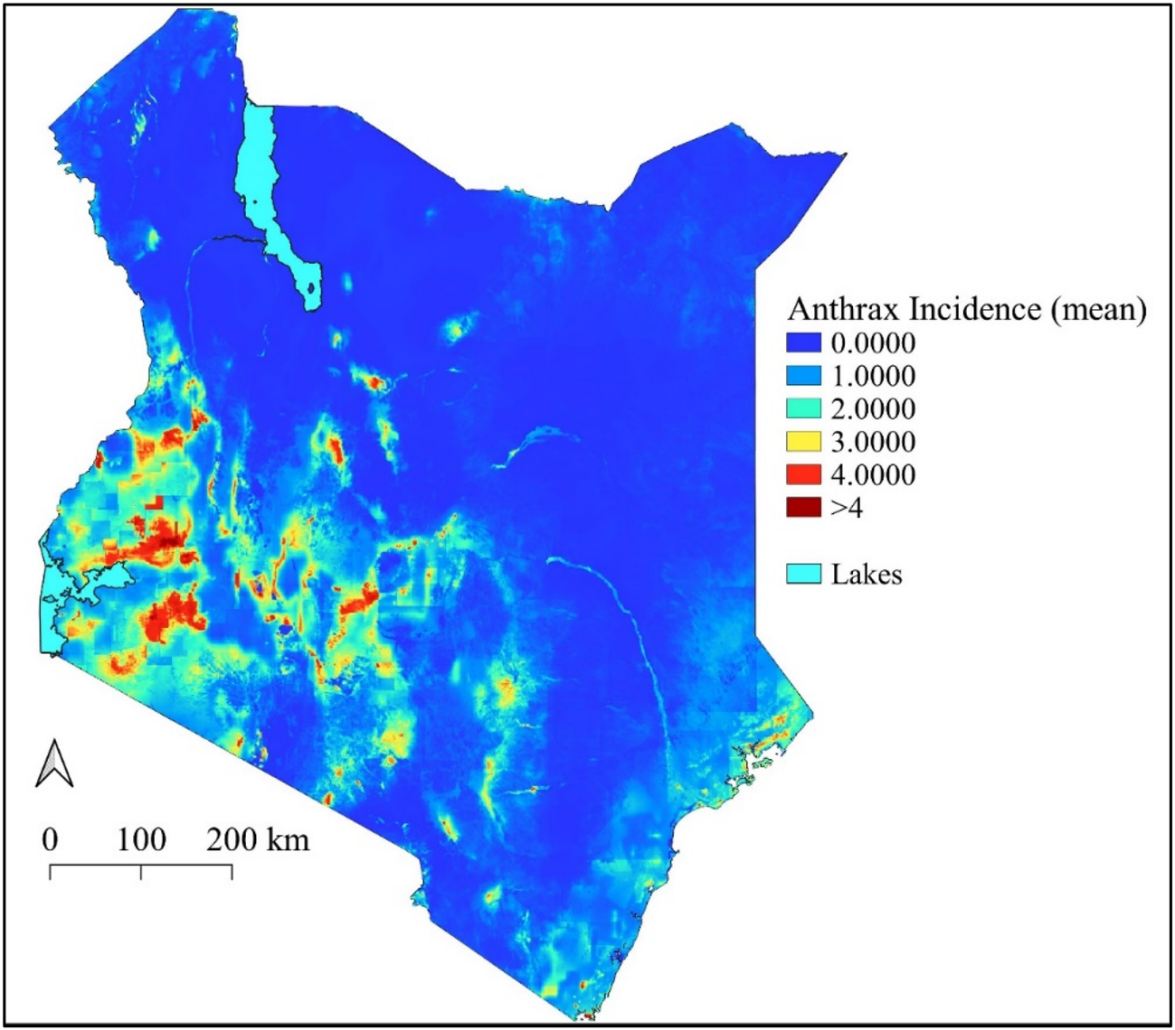
The posterior predicted mean of the incidence of anthrax disease across Kenya from livestock data (2006–2020). The scale for anthrax incidence shows colours ranging from blue to red, with blue showing areas with low incidence and warmer colours towards red showing areas with higher anthrax incidence. This map was generated using Quantum Geographical Information Systems (QGIS) v. 3.16.11 (https://www.qgis.org/en/site/forusers/download.html).

**Figure 7:**
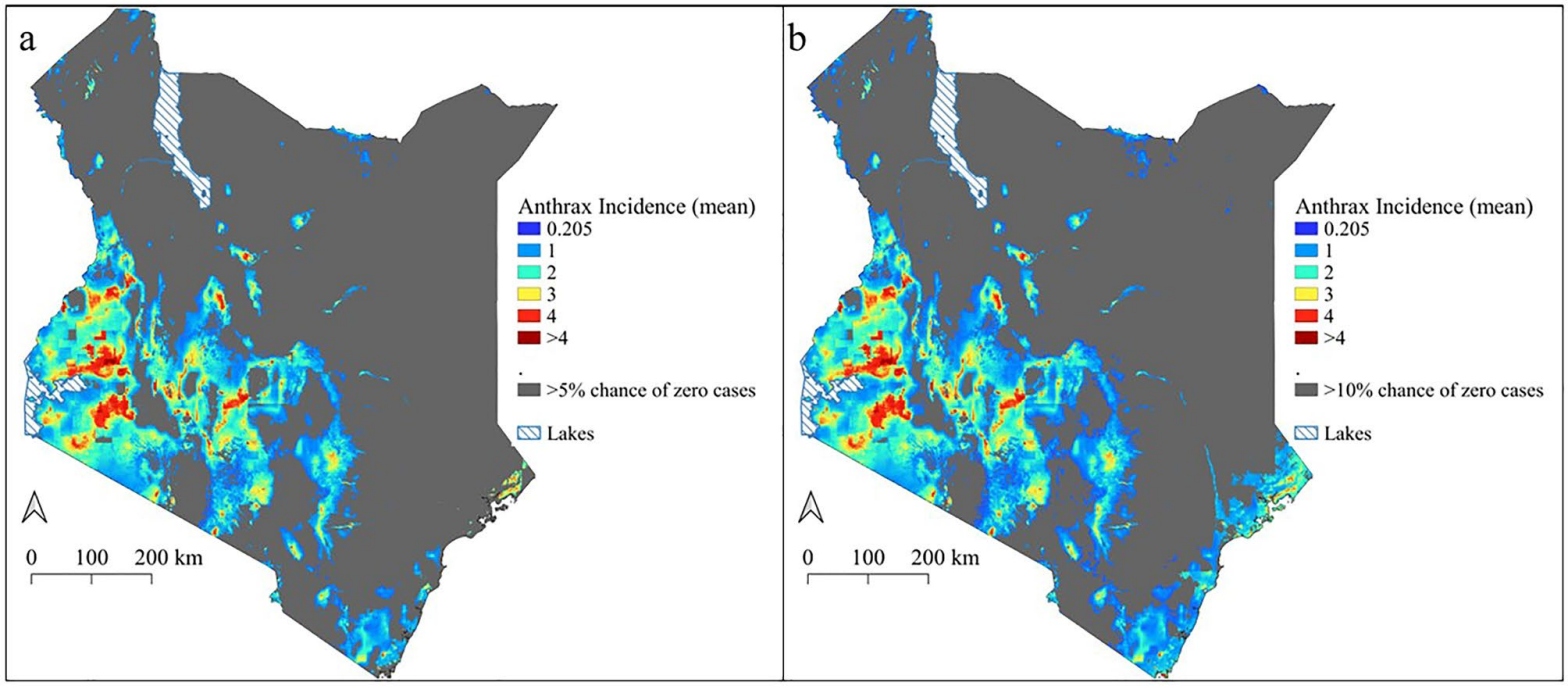
The uncertainty around the slope of the posterior predicted incidence of anthrax disease across Kenya. The grey areas show locations where greater than 5 percent (**a**) and 10 percent (**b**) of the slope of predicted incidence was below the cut-off (0.205) for positivity. We extracted the fitted density distributions of the predicted anthrax incidence and calculated the percentage/proportion of the density distribution that was lower than the positivity cut-off, then greyed out areas that had more than 5% (**a**) or 10% (**b**) of the fitted density below the cut-off. The scale for anthrax incidence shows colours ranging from blue to red, with blue showing areas with low incidence and warmer colours towards red showing areas with higher anthrax incidence. Maps generated using Quantum Geographical Information Systems (QGIS) v. 3.16.11 (https://www.qgis.org/en/site/forusers/download.html).

The spatially projected nationwide maps from the best model identified large parts of Western Kenya, the Rift-Valley, Central, and Coastal regions to be at high risk of livestock, human, and wildlife anthrax disease (Fig. 6 and Table 4), and showed the uncertainty estimates around the predictions (Fig. 7). The INLA model was able to identify locations of wildlife outbreaks withheld from the model calibration such as Shompole Wilderness, Garissa, Tsavo National Park, Meru National Reserve, Kyelu Ranch, and Kaluku, Hells Gate, Lake Nakuru National Park, Nairobi National Park, Mbagathi, and Soysambu Conservancy.

**Table 4:** Counties identified to be at high risk of livestock, human, and wildlife anthrax disease by region.

Region	Counties
Western Kenya and Rift Valley	Narok, Bomet, Kisii, Kericho, Nandi, Uasin Gishu, Kakamega, Vihiga, and Kisumu, southern regions of West Pokot and central Trans Nzoia, and Busia counties
Central	Kiambu, Muranga, southern parts of Nyeri, Kirinyaga, Nyandarua, Nakuru, and most parts of Meru, Tharaka-Nithi, and Embu
Coastal	Kwale, Kilifi, Mombasa, and Lamu
Northern	Samburu, Turkana, Marsabit, and southern regions of Garissa County

### Spatiotemporal sub-county model

#### Recent trends in anthrax surveillance in Kenya

**Figure 8:**
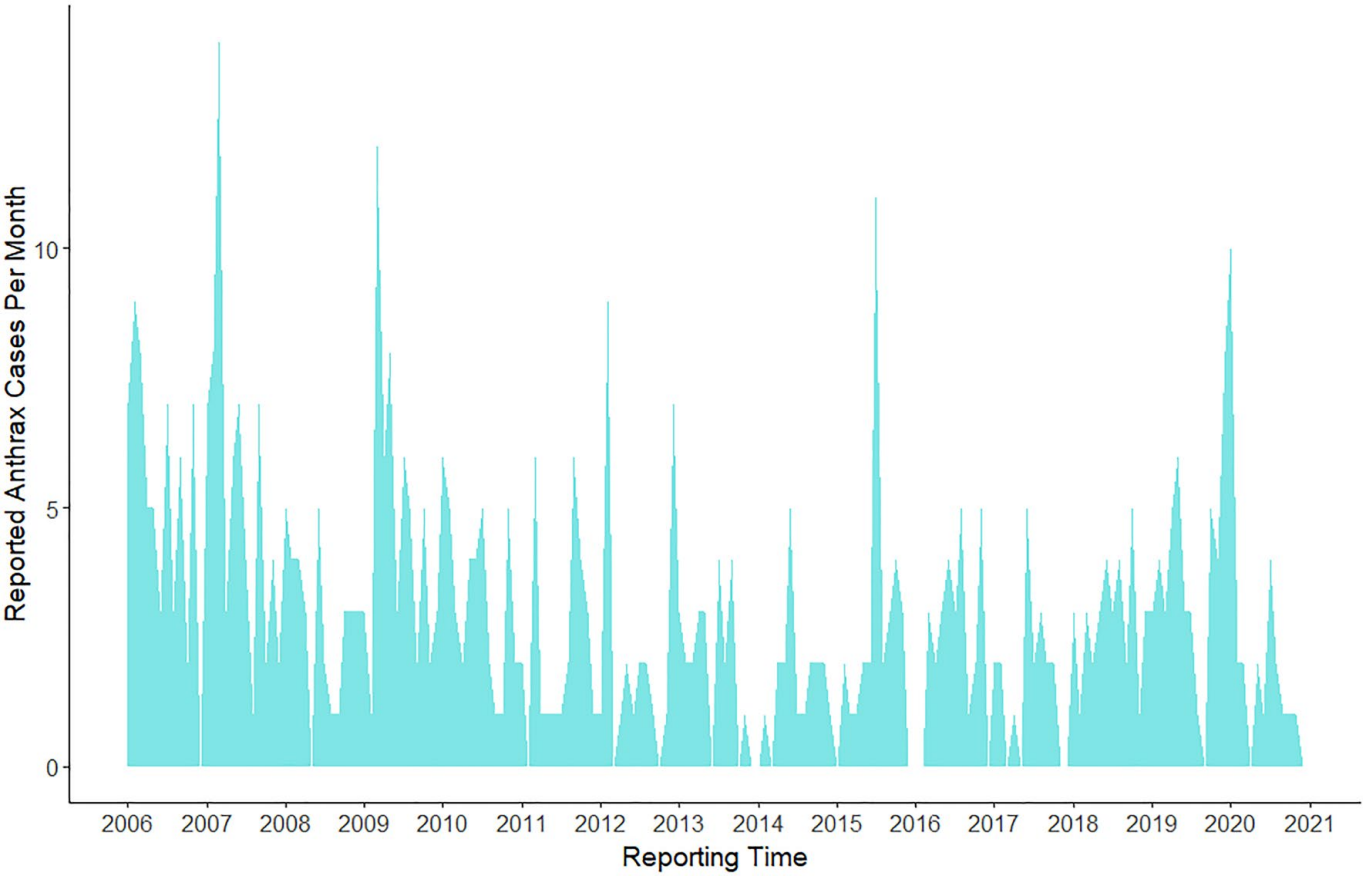
Temporal trends in country-wide livestock anthrax cases from 2006 to 2020. The polygon height illustrates the monthly total livestock cases reported across Kenya. The full anthrax case time series was assembled from the Kenya Directorate of Veterinary Services: Monthly case reports from 2006 to 2020. Full details of reporting procedures and case definitions are provided in Methods. The figure was generated using R software v. 4.1.0^28^.

**Figure 9:**
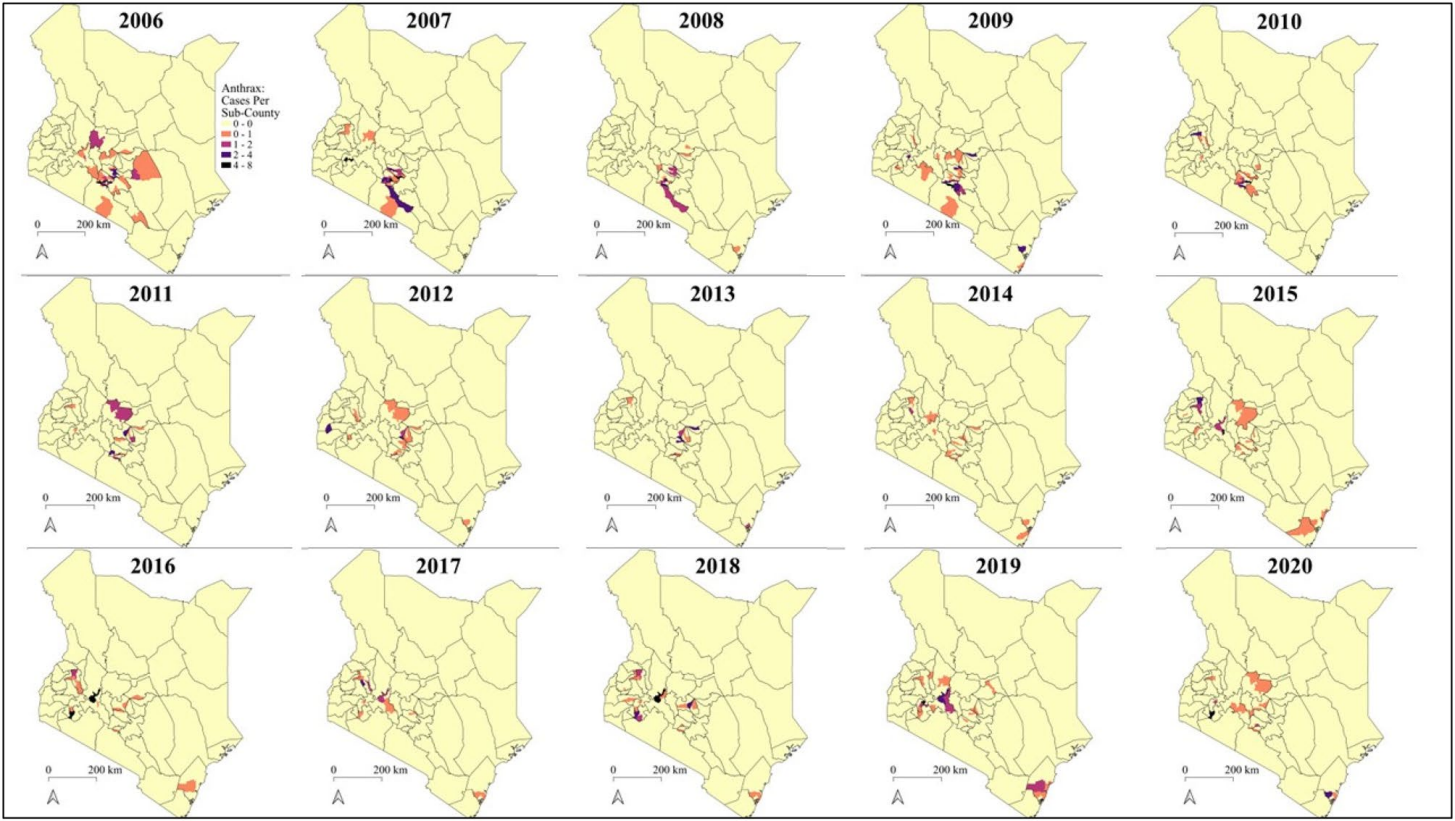
Spatiotemporal trends in confirmed livestock anthrax cases across Kenya. Maps illustrate the total reported livestock anthrax cases in each sub-county from 2009 to 2020. Map generated using Quantum Geographical Information Systems (QGIS) v. 3.16.11 (https://www.qgis.org/en/site/forusers/download.html).

We analyzed the temporal trends in confirmed and suspected livestock anthrax cases within and between years. Monthly case counts of anthrax were aggregated across the country and illustrated as aggregated monthly livestock case totals and quarterly case accumulation curves (Fig. 8). Annual peaks of anthrax cases were shown to occur from January and July, which have the dry and hot followed by the wet and cool seasons, with secondary peaks occurring between October and December which is the wet and hot season (Fig. 8). Overall temporal trends suggest that 2006–2010 appear to be distinctly different from the previous years, with markedly high peaks in confirmed livestock anthrax cases (Fig. 8). Throughout the study, 86 sub-counties in 26 of 47 counties reported confirmed livestock anthrax cases with evidence of marked spatial as well as temporal clustering (Figs. 8, 9). However, 204 sub-counties reported no confirmed livestock anthrax cases (total = 290 sub-counties; median 0 cases, mean 1.3, range 0–22). For instance, most livestock cases (~ 70%; 266/378 cases) were reported from 25 sub-counties within 13 of the 47 Kenyan counties (Bomet, Kericho, Kiambu, Kilifi, Kirinyaga, Laikipia, Mombasa, Murangá, Nairobi, Nakuru, Nyeri, Tharaka-Nithi, Uasin Gishu), with much lower incidence in northern counties.

#### Evaluating the geographical distribution and correlates of anthrax occurrence and incidence

For the spatiotemporal occurrence and incidence models, we fitted linear relationships for population density, total population, number of exotic dairy cattle, agricultural land area, and number of livestock-producing households. The models with the socio-economic variables explained significantly more of the variation within the dataset compared to the baseline (intercept only) model (occurrence ΔDIC =  − 338; incidence ΔDIC =  − 332; Table 5). The addition of the spatial random effects and the temporal trend further improved the model fit compared to the model with the covariates alone (occurrence ΔDIC =  − 583; incidence ΔDIC =  − 591;Table 5). Thus, the occurrence and incidence models with the covariates, spatial random effects, and the temporal trend were selected as the final models. Both models passed the validation checks.

**Table 5:** Model comparison of the fitted INLA occurrence and incidence models using linear associations.

Model	DIC	CPO
**Occurrence model**		
Intercept only	3923	0.112
Intercept + Tot.pop + Pop.den + Dairy.c + Agric.land + Livestock.hh	3585	0.103
Intercept + Tot.pop + Pop.den + Dairy.c + Agric.land + Livestock.hh + ɸ	3047	0.086
Intercept + Tot.pop + Pop.den + Dairy.c + Agric.land + Livestock.hh + ɸ + Ω	3002	0.085
**Incidence model**		
Intercept only	4526	0.130
Intercept + Tot.pop + Pop.den + Dairy.c + Agric.land + Livestock.hh	4194	0.120
Intercept + Tot.pop + Pop.den + Dairy.c + Agric.land + Livestock.hh + ɸ	3643	0.104
Intercept + Tot.pop + Pop.den + Dairy.c + Agric.land + Livestock.hh + ɸ + Ω	3603	0.103

Variables acronyms are *Tot.pop* Total population, *Pop.den* Population density, *Dairy.c* Exotic dairy cattle, *Agric.land* Agricultural land area, *Livestock.hh* Livestock producing households, *ɸ* Spatial random effect, *Ω* Temporal trend.

**Figure 10:**
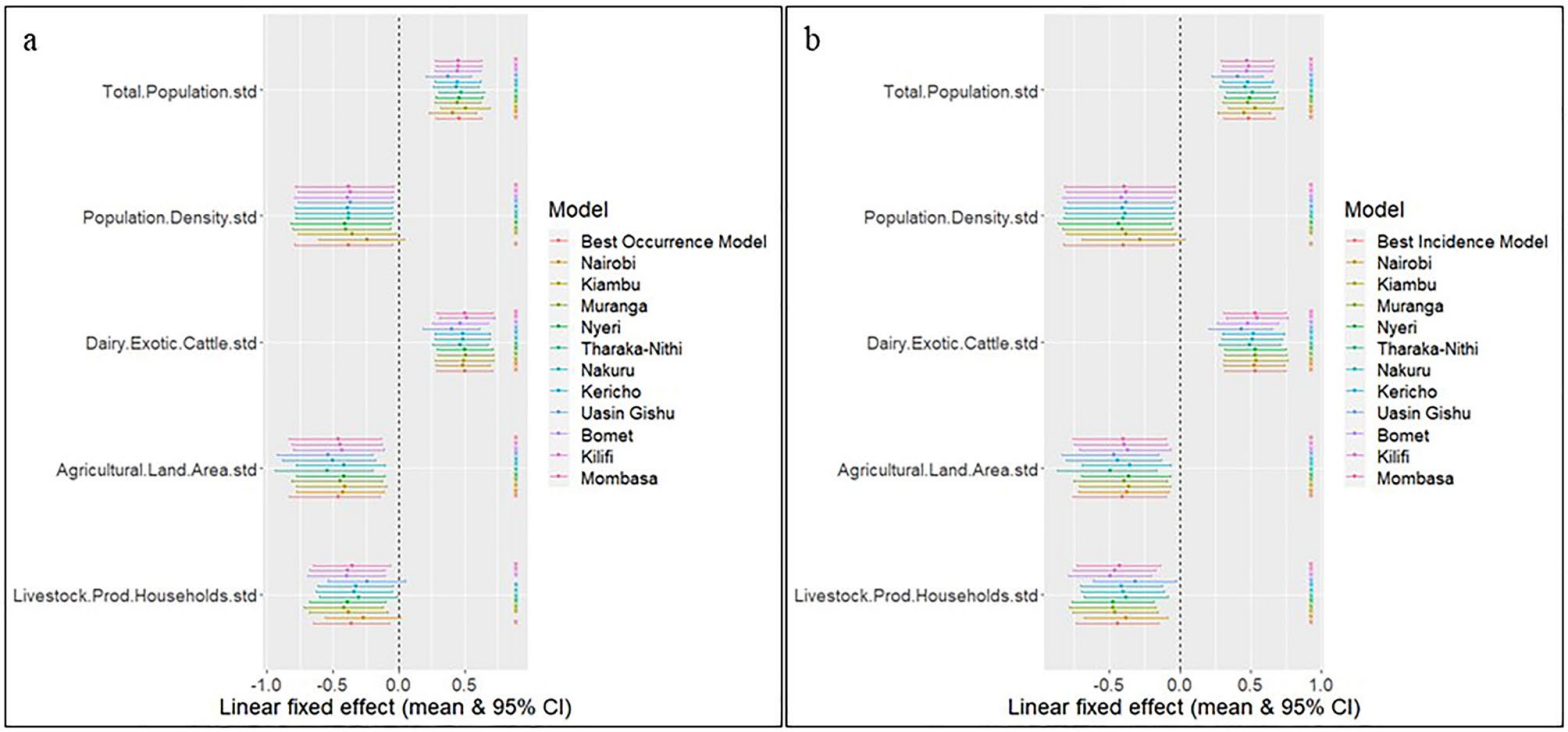
Geographical cross-validation tests for model robustness. The figures show the magnitude and direction of the fixed effect across all the holdout models to test whether the findings were overly influenced by data from any geographical location. The figure was generated using R software v. 4.1.0^28^.

To examine model robustness, we examined the sensitivity of the model outputs to geographically based cross-validation by fitting separate models, in turn, holding out high burden sub-counties from each of 11 counties that had high reported anthrax incidence (Nairobi, Kiambu, Murang’a, Nyeri, Tharaka-Nithi, Nakuru, Kericho, Uasin Gishu, Bomet, Kilifi, and Mombasa) (Table 6). The fixed effects magnitude and direction were robust across all the holdout models, showing that the findings were not overly influenced by data from any geographical location (Fig. 10).

**Table 6:** High burden sub-counties from each of 11 counties held out in turn from model fitting during geographically based cross-validation.

County	Withheld sub-counties
Nairobi	Langata, Roysambu, Westlands
Kiambu	Githunguri, Kabete, Kiambu, Kikuyu, Limuru, Ruiru, Thika Town
Murang’a	Kiharu, Maragwa
Nyeri	Mathira, Mukurweni
Tharaka-Nithi	Maara
Nakuru	Nakuru Town East, Nakuru Town West, Rongai
Kericho	Ainamoi
Uasin Gishu	Kapseret, Soy, Turbo
Bomet	Sotik
Kilifi	Kaloleni
Mombasa	Kisauni

**Figure 11:**
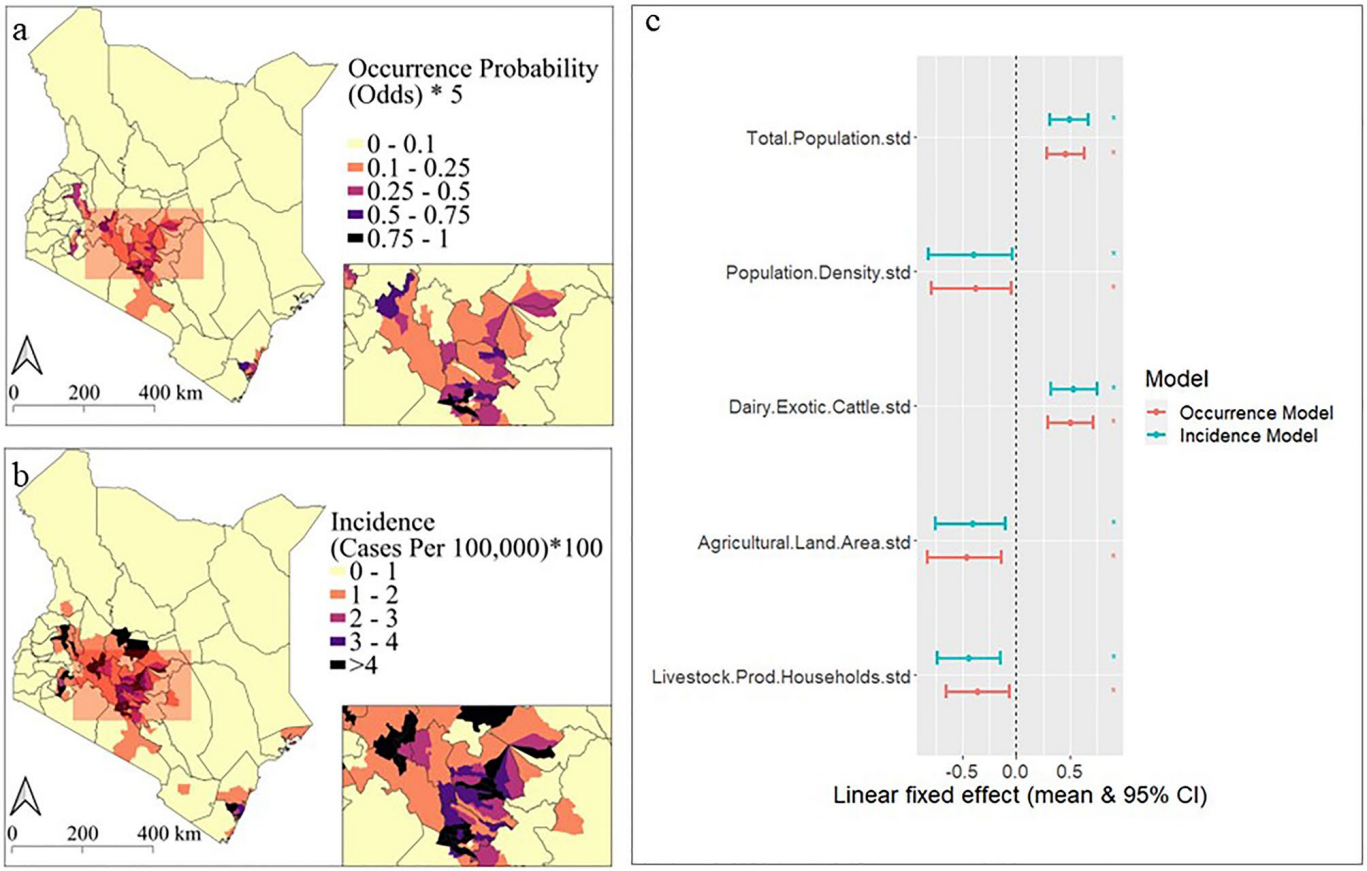
Spatial distribution and correlates of annual anthrax occurrence and incidence (2006–2020) at sub-county level across Kenya. Maps show the fitted probability of anthrax occurrence (**a**) and incidence (**b**; livestock cases per 100,000 people) for 290 sub-counties in the last quarter of 2020. The points and error bars (**c**) illustrate the parameter estimates of the linear socio-economic fixed-effects (the posterior mean estimate and the 95% credible interval) for the best-fitting models of anthrax occurrence (red) and incidence (blue) (n = 17,400 observations). The linear covariates were standardized (centered and scaled) before model fitting, such that parameters estimate the effect of 1 standard deviation change in the covariate on either the odds of occurrence or incidence. The models both included spatiotemporal random effects (sub-county per year) to incorporate spatial and temporal heterogeneity and were robust to geographical cross-validation tests (Fig. 10). Maps were generated using Quantum Geographical Information Systems (QGIS) v. 3.16.11 (https://www.qgis.org/en/site/forusers/download.html).

Both the incidence and occurrence models had positive linear effects on total population and exotic dairy cattle, and negative linear effects on population density, agricultural land area, and livestock producing households (Fig. 11c and Table 7). The spatial maps of the fitted probability of anthrax occurrence and incidence (Fig. 11a,b) suggest that large parts of Kenya (particularly Central, western, and Coastal Kenya) are suitable for anthrax transmission.

**Table 7:** The posterior means and the 95% credible intervals for the fixed effects parameters of the covariates in the spatiotemporal occurrence and incidence model.

Parameters	Occurrence model			Incidence model		
	Mean	Lower CI (2.5%)	Upper CI (97.5%)	Mean	Lower CI (2.5%)	Upper CI (97.5%)
Intercept	− 5.067	− 5.331	− 4.824	− 3.910	− 4.239	− 3.589
Total population	0.456	0.282	0.631	0.490	0.309	0.671
Population density	− 0.383	− 0.788	− 0.046	− 0.401	− 0.823	− 0.044
Dairy exotic cattle	0.500	0.289	0.711	0.533	0.314	0.752
Agricultural land area	− 0.459	− 0.830	− 0.138	− 0.405	− 0.758	− 0.100
Livestock producing households	− 0.360	− 0.650	− 0.071	− 0.440	− 0.735	− 0.148

**Figure 12:**
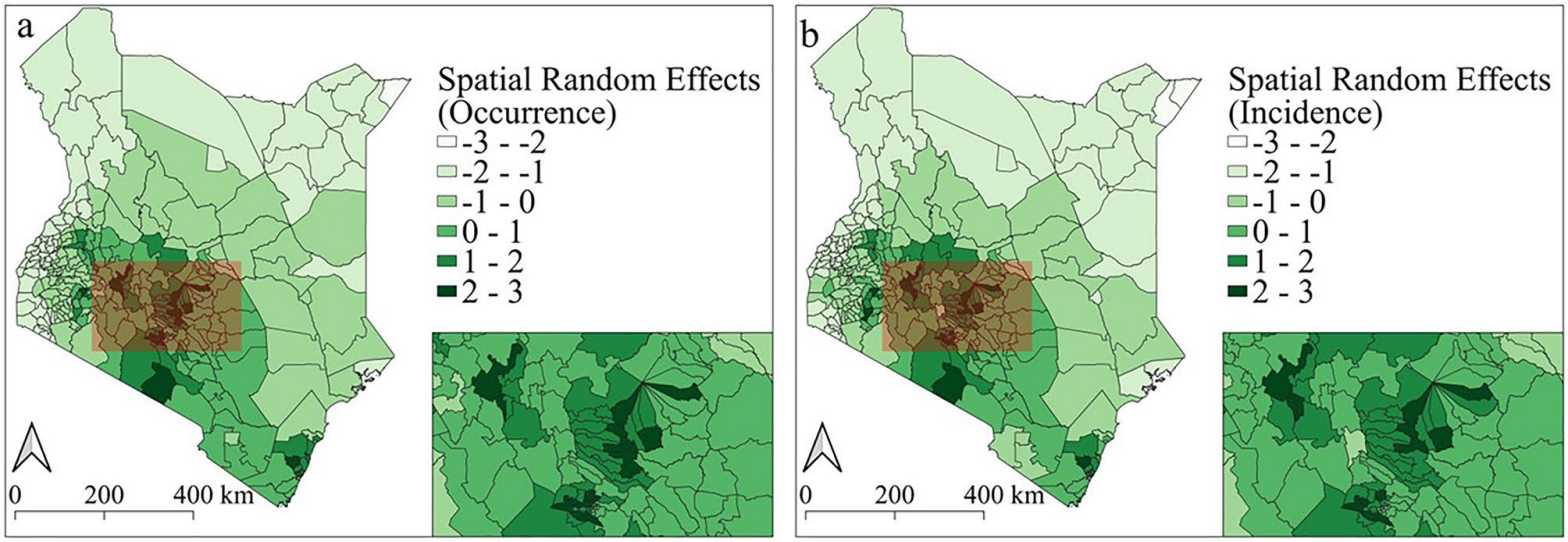
The posterior mean of the spatial random effects. The maps show the posterior mean of the spatial random effects for the occurrence (**a**) and incidence (**b**) models. Maps were generated using Quantum Geographical Information Systems (QGIS) v. 3.16.11 (https://www.qgis.org/en/site/forusers/download.html).

**Figure 13:**
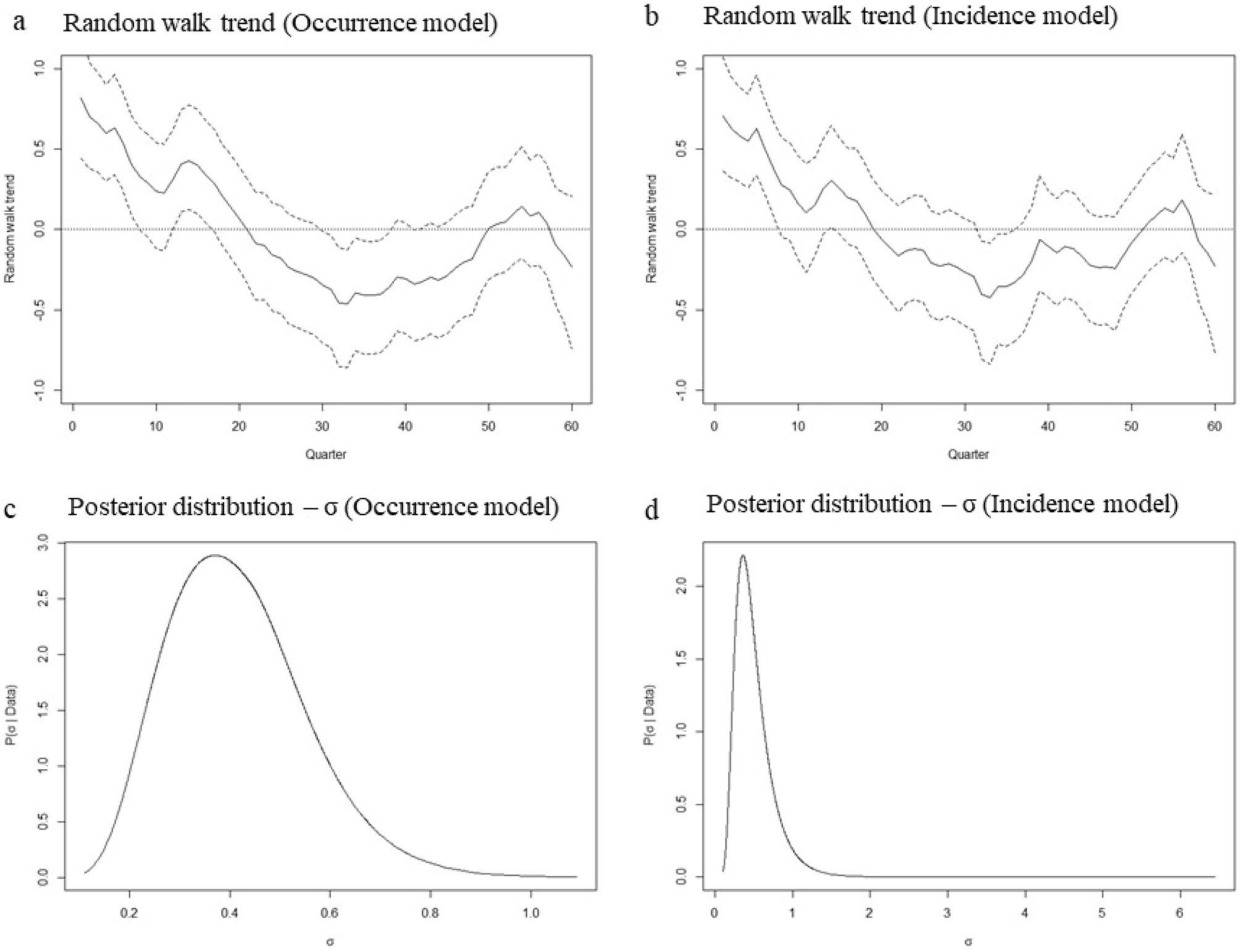
Random walk trend for the occurrence (**a**) and incidence (**b**) models. The lower panels show the marginal posterior distribution for the standard deviation (σ) hyperparameter of the random walk trend for the occurrence model (**c**) and the incidence model (**d**). The image was derived from the results of the R-INLA package^20^ implemented via R v. 4.1.0^28^.

Although the socio-economic covariates used in our models can explain a bit of the pattern of anthrax occurrence and incidence, they do not fully account for the observed spatial distribution of anthrax. There is still some residual spatial variation that is not explained by fitted covariates. Consequently, the addition of the spatial random effects (Fig. 12) and the random walk trend (Fig. 13) further improves the fit of the model. Figure 13 shows that there was a downward trend in anthrax livestock cases from 2006 to 2012 followed by a slight incline up to 2018 and then a drop again. Including this information produces a much better model fit.

## Discussion

### Spatial model

We used available livestock anthrax data from 2006 to 2020 to develop a spatial ecological model of livestock, human, and wildlife anthrax risk across Kenya using gridded environmental covariates. Our final spatial model showed that distance to water bodies had a significant negative association with anthrax incidence. This observation is consistent with past studies which have demonstrated a significant negative link between distance to water bodies and the suitability of an area for the occurrence of *B. anthracis*^15^. This is most likely linked to the fact that most animals use communal watering points, thus, there is an increased likelihood of observing anthrax outbreaks close to water bodies than further away. EVI had a strong positive association with anthrax incidence between 1367 and 5600 units. Similarly, increasing elevation was also associated with an increased incidence of anthrax disease from about 150 to 215 m.

The spatially projected nationwide maps from the best model showed large parts of Western Kenya, the Rift-Valley, Central, and Coastal regions to be at high risk of livestock, human, and wildlife anthrax disease (Fig. 3) with adequate precision (Fig. 4). The model correctly identified known locations of wildlife outbreaks within Tsavo National Park, Shompole Wilderness, Meru National Reserve, Kyelu Ranch, Hells Gate, Lake Nakuru National Park, and Nairobi National Park. Previous studies have reported wildlife and livestock anthrax outbreaks across the same areas^11,17^. The counties with the highest predicted anthrax intensity were the southern regions of West Pokot and central Trans Nzoia, and Busia counties which lie next to the eastern border of Uganda, Narok County bordering the Serengeti National Park in Tanzania, Kajiado around Mt. Kilimanjaro National Park, Taita Taveta and Makueni counties around Tsavo West National Park, and within Kitui County around Tsavo East National Park. Anthrax risk areas were also detected around Namunyak Wildlife Conservation Trust in Wamba and vast areas of Maralal and Mukawa within Samburu County. Several counties in Central Kenya were also identified as high-risk areas consistent with previously published studies^11,17,32^.

Although the results of the model suggest that many parts of Kenya are at risk of livestock, human, and wildlife anthrax, a key alarming observation is the high intensity of anthrax predicted across the Northern parts of the country, specifically across Turkana, Samburu, and Marsabit counties. Most parts of these counties are classified as arid and semi-arid land (ASAL) comprising pastoralists who are often economically and politically marginalized, lacking access to both veterinary and public health services^33^. Such services are usually unavailable in these areas due to poor communication, roads, and infrastructure^33^. Since hospitals and veterinary clinics are few or non-existent in most of these parts, these services follow the existing infrastructure and are primarily mobile-assisted by local NGOs and government-aided mobile outreaches^34^. These factors predispose the pastoralists to a greater risk of zoonotic diseases such as anthrax. The sparsity of recorded anthrax livestock outbreaks in this region could reflect the poor surveillance practices and not necessarily the absence of livestock cases.

During the 2021 Kenya One Health Conference held between December 6th and 8th, the National Strategy for Control of Anthrax in Kenya 2021–2036 was presented by Dr Augusta Kivunzya^35^. The strategy highlighted four key phases: Phase 1 focused on a preparatory and adoption phase running from 2021 to 2023 which will involve identifying the disease epidemiology and developing a structured control plan^35,36^. Although it was mentioned that the high-risk areas across Kenya would be identified from a review of past records, these records suffer from sampling bias that gives more weight to counties that have better surveillance systems^11^. The strategy also proposed focusing on anthrax reports spanning the last 5 years (2014–2019) meaning that only counties that had reported livestock anthrax cases during that time would be identified as high risk and would benefit from the pilot program which will include vaccinations and enhanced surveillance^35,36^. The first phase will also include the development of a risk map for anthrax which will be used to guide the implementation of the pilot program only within the areas that have had anthrax over the past 5 years^35,36^. Other risk areas that have not had anthrax in the past 5 years will only be classified as high-risk areas if an anthrax outbreak occurs there. In phase 2 the country will start the implementation of the strategy in high-risk areas (from 2024 to 2027) involving a nationwide livestock vaccination program, while in phase 3 the country will continue with the prevention strategy (from 2028 to 2032) with plans to review and update the national risk maps as well as obtain a countrywide vaccination coverage of 80% of the susceptible animals^35,36^. Considering this ambitious strategy, our study will provide policy makers with an idea of possible high-risk areas, particularly in the marginalized Northern Counties, where they could intensity anthrax surveillance efforts so that these counties do not miss out on the nationwide anthrax control program. Furthermore, the supply of vaccines was listed as a possible challenge and that the World Organisation for Animal Health (OIE) had offered to help by providing vaccines at a very good cost but only if Kenya presents its case as a country^35^. Considering there are 47 governments (counties) within the country, the proposed solution was to identify the various requirements of the counties and take them to OIE as a country^35^, further emphasizing the importance of boosting surveillance across all high risk areas identified in our study.

Wildlife case locations collected from KWS from 2000 to 2020 were used to validate the model performance. The model correctly predicted 15 out of the 20 wildlife anthrax case locations using the minimum training presence threshold resulting in a sensitivity of 75% (95% CI 65–75). Geographical cross-validation, performed to test the sensitivity of the model outputs by fitting a separate model holding out all the livestock data points from the coastal region of the country (n = 11), showed that the model was robust since the fixed effects magnitude and direction remained the same for the final model and the holdout model (Fig. 4). The recent continental study of the distribution of *B. anthracis* across Africa also had lower omission rates when no thinning (3.4%), 30 km thinning (10%), and 50 km thinning (5.9%) were applied to the datasets^37^. However, the models were created by randomly sampling 50% of the occurrence dataset for model calibration and the rest (50%) for model validation^37^. Random partitioning of the data into training and testing sets can inflate the performance of a model and underestimate the error in the spatial prediction evaluation^38^. Aside from the continental anthrax risk map, two additional livestock anthrax risk mapping studies were done in 2021 in Kenya^16,17^. The first article applied Boosted Regression Trees (BRTs) to model the geographical distribution of anthrax in Kenya focusing on the southern parts of the country^17^. Although the final ensemble model produced had a mean Area Under the Receiver Operating Characteristic Curve (AUC) of 0.8, the sample size used was smaller (n = 69), and the authors also restricted their model to the southern half of the country thereby limiting interpretation across the whole country^17^. The second article also applied BRTs to model the future distribution of anthrax across Kenya under various climate change scenarios^16^. The final model had a good mean training AUC (0.936; ± 0.0019) and mean testing AUC (0.929; ± 0.0039) under the current scenario^16^. However, like the continental study, the occurrence dataset was randomly partitioned by splitting 75% of the data for model training and 25% for model testing which can inflate the performance of a model^16^. Our study used an independent dataset comprising wildlife anthrax cases collected separately to validate the performance of our spatial model, as well as geographically based cross validation using a spatially distinct chunk of the livestock data. Both methods showed that the model had satisfactory precision and robustness.

#### The spatiotemporal models

The spatiotemporal occurrence and incidence models investigate the drivers of anthrax occurrence and incidence. The occurrence model investigates the factors that determine whether an outbreak occurs or not (presence or absence), while the incidence model investigates what determines the severity of an outbreak (number of livestock cases) after it occurs. Both are equally important because they provide insight that can help policy makers to design interventions to prevent an outbreak from occurring or to reduce the severity if it occurs. Both models show that livestock anthrax risk is strongly influenced by an increasing total human population which may increase demand for meat and hence the host population sizes, animal-human contact, access to healthcare, and anthrax awareness. Similarly, there is a substantial positive association with the number of exotic dairy cattle which can also influence host population sizes and human contact. The negative association between livestock anthrax risk and population density could be linked to increased risk in rural areas, which are less densely packed and have reduced access to healthcare and anthrax awareness programs and have more livestock in general compared to urban areas. The negative effect of increasing the number livestock producing households on livestock anthrax risk could be due to increased awareness of anthrax in areas with greater numbers of livestock farmers. Similarly, this might also explain the negative association between agricultural land area and anthrax occurrence. These findings were robust to geographically based cross-validation and were not overly influenced by data from any geographical location (Fig. 9).

The spatial map of the fitted probability of anthrax occurrence and incidence (Fig. 10a,b) shows that large parts of Central, Western, and Coastal Kenya appear suitable for anthrax transmission. One explanation for the findings could be that the geographical distribution of anthrax is mainly influenced by the surveillance effort and unreported livestock cases are more present everywhere than recognized. This is backed by the fact that *B. anthracis* spores can survive across diverse geographical regions even under severe environmental stress. In addition, since anthrax prevalence in livestock can change over tiny geographical scales, such as between sub-counties, it is likely that livestock anthrax risk is highly localized. For instance, changes in the host populations, spore dispersal, immunity, and infection dynamics, could cause significant variations in the risk of spillover across space and time. There is currently very limited information on the prevalence of livestock anthrax vaccination in Kenya because vaccination is often performed as a reaction to an outbreak^36^. However, the recent development of the “National Strategy for the Prevention and Control on Anthrax in Kenya in Humans and Animals in Kenya (2021–2036)” will provide systematically collected data on routine livestock anthrax vaccination coverage^36^. To detect underreported areas at risk of livestock, human, and wildlife anthrax and target prevention interventions, future research outside the known endemic areas is necessary to investigate the unmeasured environmental and social factors driving anthrax risk e.g., high clinical and public knowledge and awareness, access to healthcare, agricultural activities, host immunity dynamics, or animal movement patterns.

## Conclusion

Overall, the extent of the anthrax endemic area seems to be well defined by the various ecological and socioeconomic conditions that drive human-host contact. Anthrax incidence in endemic regions is greater in areas with a higher total human population and numbers of exotic dairy cattle, suggesting that the socioeconomic variables influencing human exposure to anthrax are important drivers of disease risk. Public health programs aimed at reducing human-animal contact, improving access to healthcare, and increasing anthrax awareness, may have a positive effect in terms of reducing anthrax occurrence and incidence. By accounting for spatial dynamics within our observed data we have demonstrated an approach that is useful for studies that have surveillance data with spatial and temporal structural dependencies^39^. Bayesian models based on INLA are more flexible, fast, and easy to interpret and implement even for non-experts^18^. The risk map we produce here for anthrax can support the planning of surveillance and prevention campaigns that can reduce the catastrophic impacts of disease livestock and human outbreaks particularly in marginalized pastoralist communities which are disproportionately affected.

## Data Availability

The datasets and R code supporting the conclusions of this article are available in the GitHub repository, https://github.com/spatialmodels/Kenyan_anthrax_model.
